# Non‐Autoimmune Hyperthyroidism Associated With a Novel Germline Mutation (D633N) in the TSH Receptor

**DOI:** 10.1155/crie/3274433

**Published:** 2026-06-26

**Authors:** Sayuri Mori, Yoshinori Osaki, Hiroshi Fukazawa, Takaaki Matsuda, Yuki Murayama, Yoko Sugano, Hitoshi Iwasaki, Bryan J. Mathis, Motohiro Sekiya, Hitoshi Shimano

**Affiliations:** ^1^ Department of Endocrinology and Metabolism, University of Tsukuba Hospital, 2-1-1 Amakubo, Tsukuba, 305–8576, Ibaraki, Japan, tsukuba.ac.jp; ^2^ Department of Endocrinology and Metabolism, Institute of Medicine, University of Tsukuba, 1-1-1 Tennodai, Tsukuba, 305–8575, Ibaraki, Japan, tsukuba.ac.jp; ^3^ Department of Health Care Center Yurigaoka, Mito Chuo Hospital, 1136–1 Rokutanda-Chou, Mito, 310–0015, Ibaraki, Japan; ^4^ Tsukuba Clinical Research and Development Organization (T-CReDO), University of Tsukuba, 2-1-1 Amakubo, Tsukuba, 305–8576, Ibaraki, Japan, tsukuba.ac.jp; ^5^ Department of Cardiovascular Surgery, Institute of Medicine, University of Tsukuba, 2-1-1 Amakubo, Tsukuba, 305-8576, Ibaraki, Japan, tsukuba.ac.jp

**Keywords:** case report, familial thyrotoxicosis, nonautoimmune hyperthyroidism, TSHR germline mutations

## Abstract

Nonautoimmune hyperthyroidism resulting from an activating germline thyroid‐stimulating hormone receptor (TSHR) gene mutation is a rare condition that often presents a diagnostic challenge. We report the case of a 75‐year‐old woman with a 20‐year history of goiter and subclinical hyperthyroidism who presented with overt thyrotoxicosis. Laboratory tests confirmed hyperthyroidism with suppressed TSH, whereas thyrotropin receptor antibody (TRAb) and thyroid‐stimulating antibody (TSAb) were negative, making it difficult to distinguish from toxic multinodular goiter. Initial management with potassium iodide was ineffective. Subsequent radioactive iodine (RI) therapy was complicated by severe destructive thyroiditis with worsening thyrotoxicosis and heart failure. Following hormonal control with methimazole (MMI) and dexamethasone, a total thyroidectomy was performed for definitive management, successfully normalizing thyroid function. Histopathology revealed an adenomatous goiter. Postoperative genetic analysis using peripheral blood DNA, prompted by the atypical clinical course, identified a novel heterozygous germline mutation in the TSHR gene: c.1897G > A (p.D633N). This case expands the spectrum of activating TSHR mutations causing nonautoimmune hyperthyroidism. Functional analysis demonstrated modest constitutive activity of the variant, which may explain the unusually late clinical onset. This report underscores the importance of considering nonautoimmune hyperthyroidism, including nonautoimmune hyperthyroidism, in patients with antibody‐negative hyperthyroidism, even in elderly individuals without a known family history.

## 1. Introduction

Thyrotoxicosis may result from several conditions, including Graves’ disease, painless thyroiditis, and toxic multinodular goiter. Graves’ disease is the most common cause of hyperthyroidism. However, a subset of patients present with antibody‐negative hyperthyroidism. Previous studies have reported that ~4.5% of patients in selected cohorts with hyperthyroidism accompanied by diffuse goiter and negative thyrotropin receptor antibodies (TRAbs) harbor activating thyroid‐stimulating hormone receptor (TSHR) variants [1]. These disorders are collectively referred to as nonautoimmune hyperthyroidism and may occur in either sporadic or familial forms.

The TSHR, located on the basolateral membrane of thyroid follicular cells, is a G‐protein‐coupled receptor (GPCR) that primarily couples to Gs and Gq proteins to regulate thyroid hormone synthesis, secretion, and follicular cell proliferation. Upon binding of TSH, the receptor activates intracellular signaling pathways, primarily through Gs‐mediated stimulation of cyclic adenosine monophosphate (cAMP) and, to a lesser extent, through Gq‐mediated phosphoinositide signaling [2, 3]. Persistent activation of these pathways due to activating TSHR variants leads to excessive thyroid hormone production and thyroid enlargement.

Activating germline TSHR variants cause nonautoimmune hyperthyroidism, which may present with diffuse goiter, negative thyroid autoantibodies, and the absence of autoimmune manifestations such as thyroid eye disease or pretibial myxedema [4]. Although patients may respond biochemically to antithyroid drugs (ATDs), long‐term remission after drug withdrawal is uncommon, and definitive treatment is often required.

## 2. Case Presentation

**Figure 1:**
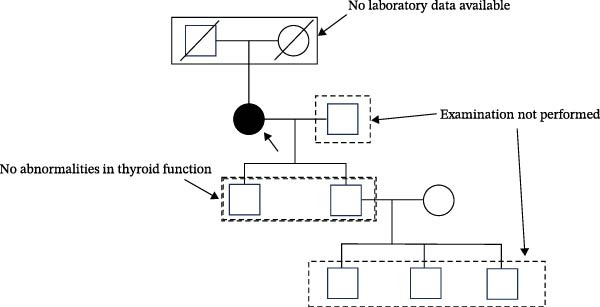
Family pedigree. The sons had normal thyroid hormone levels. No data are available on her parents (deceased before referral), and her grandchildren had not undergone testing.

**Figure 2:**
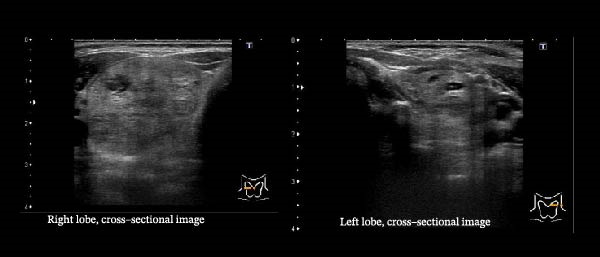
Thyroid ultrasound examination. Isthmus thickness: 8 mm, right lobe: 24 mm × 27 mm × 67 mm, and left lobe: 28 mm × 21 mm × 69 mm. Multiple adenomatous nodules occupied the entire lobe.

Our patient was a 75‐year‐old Japanese woman with persistently low TSH and an adenomatous goiter since age 53. She became aware of a thyroid mass at 73 years of age, and at 74 years of age, thyrotoxicosis was diagnosed (TSH < 0.005, FT4 1.95) before referral to our department. She had no significant past medical history, no regular medications, and no family history of thyroid disease (Figure 1). At the time of diagnosis, the patient was largely asymptomatic except for thyroid enlargement. She did not report weight loss, palpitations, fatigue, anorexia, or any other classical symptoms of hyperthyroidism. Physical examination revealed a firm, rubber‐like diffuse goiter without tenderness. The goiter remained painless throughout the clinical course, and no local compressive symptoms such as dysphagia, dyspnea, or hoarseness were reported. Marked enlargement of both thyroid lobes was noted, and ultrasound was unable to distinguish between diffuse thyroid enlargement and multiple adenomatous nodules occupying the entire lobe (Figure 2). Estimated thyroid volume was 58.7 mL [5]. A mild increase in thyroid blood flow was observed, and laboratory tests showed TSH < 0.005 μU/mL (0.5–5), FT3 3.9 pg/mL (2.3–4), and FT4 1.52 ng/dL (0.9–1.7), with negative TRAb, thyroid‐stimulating antibody (TSAb), TgAb, and TPOAb (Table 1). ^123^I thyroid scanning showed diffuse uptake with a cold spot in the lower portion of the left lobe (uptake rate was 20.2% at 24 h).

**Table 1:** Initial laboratory values.

Test	Result	Reference range
Complete blood count		
White blood cells (µL)	5800	4000–9000
Hemoglobin (g/dL)	11.8	14.0–18.0
Platelet (10^4^/µL)	21.7	15.0–35.0
Biochemistry		
Total protein (g/dL)	6.8	6.7–8.3
Albumin (g/dL)	3.9	3.8–5.3
AST (U/L)	25	8–38
ALT (U/L)	29	4–44
ALP (U/L)	261	104–338
GGT (IU/L)	88	8–38
T‐Bil (mg/dL)	0.5	0.3–1.2
CK (U/L)	87	42–150
BUN (mg/dL)	17.0	8–20
Creatinine (mg/dL)	0.63	0.47–0.79
Plasma glucose (mg/dL)	94	70–199
Sodium (mmol/L)	142	135–147
Potassium (mmol/L)	4.2	3.6–5.0
Chloride (mmol/L)	107	98–108
Calcium (mg/dL)	9.6	8.6–10.1
LDL‐C (mg/dL)	66	65–139
C‐reactive protein (mg/dL)	0.08	0.0–0.2
Endocrine examination		
TSH (μU/mL)	<0.005	0.5–5.0
Free T3 (pg/mL)	3.9	2.3–4.0
Free T4 (ng/dL)	1.52	0.9–1.7
Thyroglobulin (ng/mL)	325	0–39.7
TRAb (IU/mL)	<0.8	<1.9
TSAb (%)	114	0–120
TPO‐Ab (IU/mL)	3.3	0–9.4
Tg‐Ab (IU/mL)	<10.0	0–54.6
hCG (mIU/mL)	1.5	0–0.5

Abbreviations: ALP, alkaline phosphatase; ALT, alanine aminotransferase; AST, aspartate aminotransferase; BUN, blood urea nitrogen; CK, creatine kinase; FT3, free triiodothyronine; FT4, free thyroxine; GGT, gamma glutaryl transferase; hCG, human chorionic gonadotropin; LDL‐C, low‐density lipoprotein cholesterol; Tg‐Ab, antithyroglobulin antibody; TPO‐Ab, antithyroid peroxidase antibody; TRAb, thyrotropin receptor antibody; TSAb, thyroid‐stimulating antibody; TSH, thyroid‐stimulating hormone.

**Figure 3:**
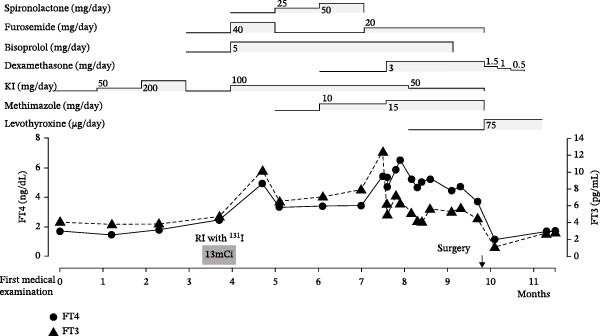
Clinical course of thyroid hormone levels over time. The horizontal axis shows the time in months from the initial visit, and the vertical axis shows thyroid hormone levels. KI, potassium iodide; RI, radioiodine therapy.

**Figure 4:**
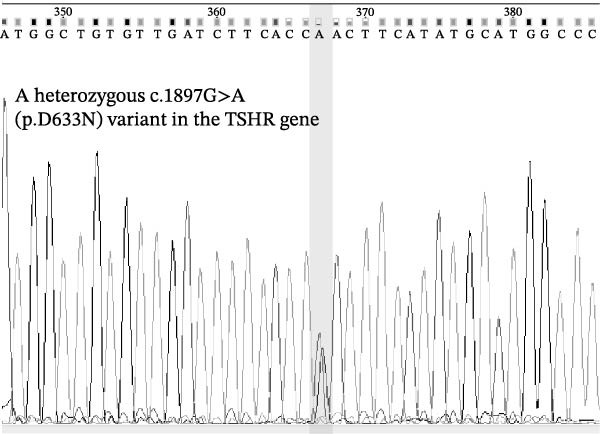
Sequencing analysis results of the TSHR gene in genomic DNA. A heterozygous guanine to adenine transition at position 1897 (arrow) in the proband. TSHR, thyroid‐stimulating hormone receptor.

Based on these findings, toxic multinodular goiter or nonautoimmune hyperthyroidism was suspected, and treatment was initiated in consideration of the suppressed TSH level (<0.1 μU/mL) and the associated cardiovascular risk. Although ATD therapy was considered, potassium iodide was started because the patient was very concerned about the adverse effects of ATDs. After 4 months, her thyroid hormone levels were not improved. Since her overall condition remained stable and concerns about adverse ATD effects persisted, she received radioactive iodine (RI) therapy. Two weeks after RI therapy, a decline in the FT3/FT4 ratio occurred, but a marked rise in thyroid hormone levels compared with pretreatment values suggested destructive thyroiditis (TSH < 0.005 μU/mL, FT4 4.92 ng/dL, and FT3 10.2 pg/mL). Two months later, edema developed. Despite initiation of methimazole (MMI) at 10 mg, the patient showed limited improvement. The dose of MMI was increased to 15 mg, and dexamethasone 3 mg was added. After 3 months, there was some improvement in destructive thyroiditis, but it had not completely normalized (FT3 5.1 pg/mL and FT4 5.22 ng/dL). Because prolonged dexamethasone administration was likely and the patient had also developed heart failure, a total thyroidectomy was performed. She underwent a total thyroidectomy nearly 9 months after initial presentation, after which the dexamethasone dose was reduced to 1.5 mg, and levothyroxine at 75 µg/day was started. Dexamethasone was gradually tapered at a rate of 0.5 mg every 2 weeks. No iatrogenic adrenal insufficiency occurred during or after the tapering process. Her thyroid function normalized (Figure 3). Pathological examination revealed an adenomatous goiter. Considering the mild clinical course and the occurrence of destructive thyroiditis, toxic multinodular goiter was considered unlikely. With the patient’s consent, genetic testing was performed at Dokkyo Medical University to investigate other possible causes. Genetic analysis was performed as part of a research‐based investigation after obtaining written informed consent from the patient. Genetic analysis using DNA extracted from peripheral blood identified a heterozygous TSHR c.1897G > A (p.D633N) variant (Figure 4), confirming a germline mutation. D633 is located within a known activating hotspot of the receptor, and other substitutions at this residue have been reported in nonautoimmune hyperthyroidism. According to ACMG 2015 criteria (PM1, PM5, PP2, and PP4), the variant was classified as a likely pathogenic activating TSHR mutation consistent with the patient’s phenotype.

**Figure 5:**
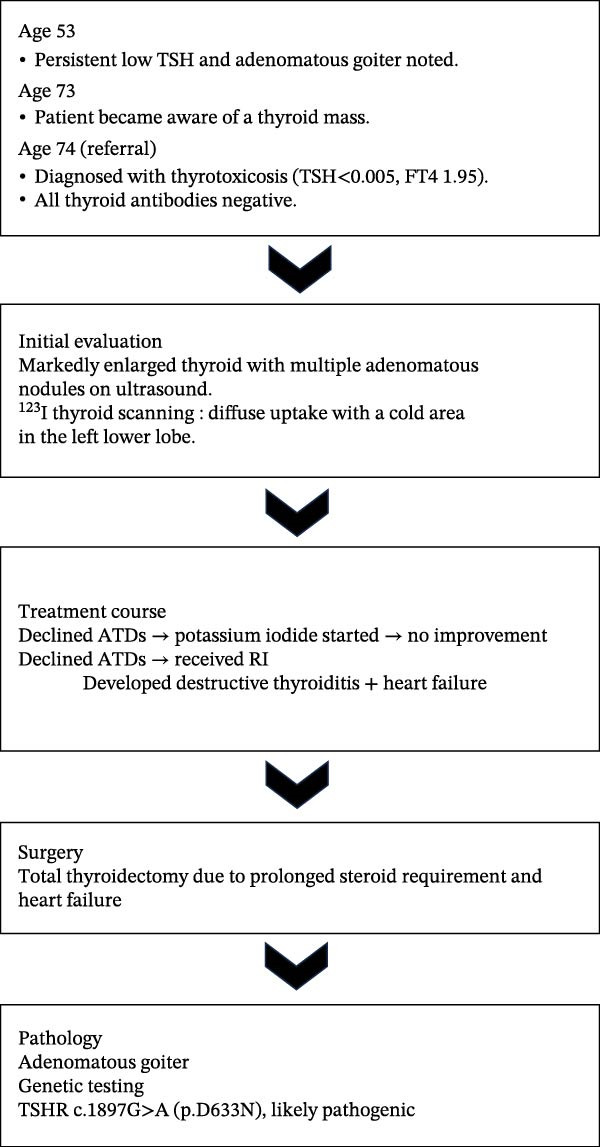
Summary flowchart of the patient’s clinical course and diagnostic pathway. Key clinical events, treatments, and the identification of the TSHR D633N variant are shown.

The patient’s long‐term clinical course, treatment timeline, and diagnostic process are summarized in Figure 5.

## 3. Experimental Methods

### 3.1. Plasmid Construction

The wild‐type human TSHR plasmid was a gift from Bryan Roth (Addgene plasmid #66518), and the wild‐type TSHR construct was cloned into the pcDNA3.1 plasmid vector. Next, mutagenesis PCR was performed using Phusion Plus DNA Polymerase (Thermo, F630S) with the following primer pairs, according to the product instructions, to create a plasmid containing the D633N, D633E, D633Y, or V656F mutations in the wild‐type TSHR plasmid. All subcloned fragments were confirmed by sequencing.

D633N

Fw: TTTCACGAATTTTATCTGTATGGCAC

Rv: ATAAAATTCGTGAAAATGAGCACGGC

D633E

Fw: TCACGGAGTTTATCTGTATGGCACCT

Rv: AGATAAACTCCGTGAAAATGAGCACG

D633Y

Fw: TTTCACGTATTTTATCTGTATGGCAC

Rv: ATAAAATACGTGAAAATGAGCACGGC

V656F

Fw: GATCACCTTTTCCAATAGCAAGATCC

Rv: TTGGAAAAGGTGATCAAAGGTTTGTT

### 3.2. cAMP Accumulation Assay

Transient expression of the wild‐type and mutant TSHR was performed in 96‐well plates (1 × 10^4^ COS‐7 cells/well) using the FuGENE HD Transfection Reagent (Promega, E2311) according to the manufacturer’s instructions.

Forty‐eight hours after transfection, the cells were washed once with PBS and then preincubated in the serum‐free Dulbecco’s modified Eagle’s medium (DMEM) containing 500 μM of 3‐isobutyl‐1‐methylxanthine (Selleck, S5836) for 1.5 h at 37°C.

Thereafter, the cAMP content of the cell was measured with the cAMP‐Glo Max Assay (Promega, V1681) according to the manufacturer’s instructions. Results were presented as fold changes relative to the wild‐type TSHR group.

## 4. Results

**Figure 6:**
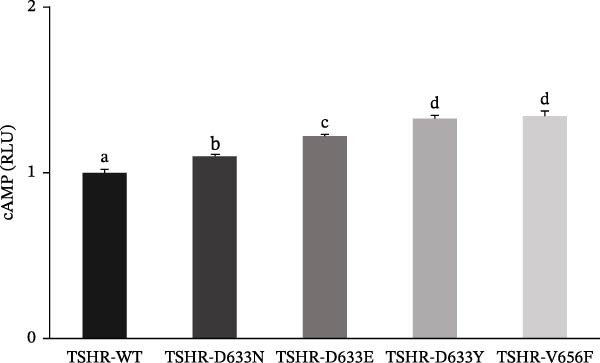
Comparison of cAMP production across TSHR variants. Data are presented as relative light units (RLU) normalized to WT (WT = 1.00) and shown as mean ± SEM of three independent experiments (*n* = 3). All variants showed significantly higher basal cAMP activity compared with WT (*p* < 0.05). No significant difference was observed between TSHR‐D633Y and TSHR‐V656F.

Basal cAMP levels were quantified in COS‐7 cells transiently expressing each TSHR construct, and the results are summarized in Figure 6. Basal activity was expressed as relative light units (RLU) normalized to wild‐type TSHR (WT = 1.00). Each experiment was performed in triplicate (*n* = 3 per construct). The relative cAMP levels were as follows: WT, 1.00 RLU; D633N, 1.10 RLU; D633E, 1.22 RLU; D633Y, 1.33 RLU; and V656F, 1.34 RLU. One‐way ANOVA followed by Tukey’s HSD test revealed significant differences among the TSHR constructs (*p* < 0.05), with D633N, D633E, D633Y, and V656F exhibiting significantly higher basal activity than WT. The known activating mutant V656F exhibited the highest basal activity among the constructs tested.

## 5. Discussion

In this case, overt hyperthyroidism with diffuse goiter and the absence of TRAb and TSAb made it difficult to distinguish between toxic multinodular goiter and nonautoimmune hyperthyroidism. Ultimately, a total thyroidectomy was performed, and pathological examination of the removed thyroid tissue revealed an adenomatous goiter. Genetic analysis using peripheral blood DNA identified a heterozygous TSHR variant, c.1897G > A (p.D633N). The D633N mutation is new and has not been previously reported in the literature. This variant is located in the sixth transmembrane helix (TM6) of the TSHR, an area known to play a key role in receptor activation [6] and may contribute to diffuse goiter and thyrotoxicosis.

In patients with subclinical or overt hyperthyroidism accompanied by diffuse goiter and negative TRAb and TSAb, nonautoimmune hyperthyroidism caused by activating TSHR variants should be considered. Several cases have been reported in Japan in which antibody‐negative subclinical hyperthyroidism or pregnancy‐related transient hyperthyroidism persisted and was ultimately diagnosed as nonautoimmune hyperthyroidism or other TSHR‐activating conditions [7, 8].

Nonautoimmune hyperthyroidism is a form of hyperthyroidism caused by germline‐activating mutations in the TSHR gene. Previous studies have reported that activating TSHR mutations are present in ~4.5% of patients with subclinical or overt hyperthyroidism and diffuse goiter who test negative for TRAb [1]. Clinical presentations vary in the severity of thyrotoxicosis and in the presence or absence of goiter [9], but nonautoimmune hyperthyroidism should be suspected when hyperthyroidism persists despite negative thyroid autoantibodies [10]. Reported activating TSHR mutations associated with nonautoimmune hyperthyroidism are summarized in Table 2.

**Table 2:** Reported activating TSHR mutations associated with non‐autoimmune hyperthyroidism.

Mutation	Location	Type	Phenotype	Onset	Basal cAMP activity	Reference
p.D633E	TM6	Germline	Neonatal hyperthyroidism with diffuse goiter	Neonatal	Markedly increased	[11]
p.D633Y	TM6	Germline	Infantile hyperthyroidism	Infancy	Markedly increased	[12]
p.V656F	ECL3	Germline	Neonatal hyperthyroidism	Neonatal	Moderately increased	[13]
p.I640V	TM6	Germline	Variable hyperthyroidism with goiter	Childhood‐adult	Moderately increased	[14]
p.D633N	TM6	Germline	Late‐onset hyperthyroidism	75 years	Moderately increased	Present case

Abbreviations: cAMP, cyclic adenosine monophosphate; ECL, extracellular loop; TM, transmembrane domain.

In the present case, a germline TSHR mutation involving codon 633 was identified. Activating mutations at this residue (e.g., D633E [11] and D633Y [12]) represent a well‐recognized hotspot within the sixth transmembrane domain of the receptor and have also been repeatedly reported as somatic mutations in toxic thyroid adenomas [15, 16]. To evaluate the functional consequences of this novel variant, we performed functional analyses comparing D633N with previously reported activating variants, including V656F, as well as D633E and D633Y.

The relatively modest constitutive activity of the D633N variant observed in the cAMP assay may partly explain the relatively late clinical onset in this patient. Compared with previously reported activating TSHR mutations associated with early‐onset disease, the lower basal signaling activity of this variant may result in mild and slowly progressive thyroid stimulation. Over time, gradual thyroid enlargement may eventually lead to clinically overt hyperthyroidism. Previous reports have suggested that activating TSHR mutations may present later in life, particularly when the constitutive activity of the receptor is relatively modest, leading to slowly progressive thyroid stimulation over time [8].

Residue D633 is known to maintain the receptor in its inactive conformation through interaction with N674 in TM7 [17]. The D633N variant exhibited ~1.3‐fold higher basal cAMP activity compared with wild‐type TSHR while maintaining TSH‐stimulated activity nearly identical to that of the wild‐type receptor [6]. Other substitutions at this position, such as D633F or D633W, have been reported to produce markedly stronger receptor activation [6]. Because the constitutive activity of the D633N variant appears relatively modest, stimulation of thyroid hormone production may have been mild and slowly progressive.

However, the phenotypic variability observed in patients with activating TSHR mutations suggests that receptor activity alone does not fully determine the disease severity. In a report describing the TSHR I640V mutation, the severity of goiter and hyperthyroidism varied among individuals carrying the same pathogenic variant, and nonproband relatives often exhibited particularly mild forms of disease [14]. Other studies have also suggested that epigenetic factors, IGF‐1 levels, iodine intake, and other environmental influences may contribute to the heterogeneous clinical presentation of hyperthyroidism and goiter in individuals with TSHR mutations [18].

**Figure 7:**
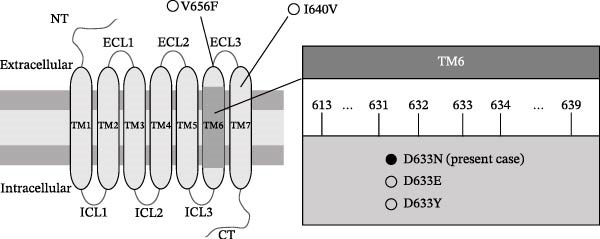
Location of activating TSHR mutations analyzed in this study. Schematic representation of the thyrotropin receptor (TSHR) topology highlighting the location of the D633N mutation identified in the present case. The mutation is located within the sixth transmembrane domain (TM6), a region known to harbor activating variants. Previously reported activating mutations affecting the same residue (D633E and D633Y) and nearby activating variants (I640V and V656F) are also shown for comparison.

Further evidence supporting this concept comes from reports of other activating TSHR variants. For example, the V656F mutation, located in the third extracellular loop of TSHR, has been reported to cause neonatal‐onset nonautoimmune hyperthyroidism despite only moderate increases in basal cAMP activity in vitro [13]. In addition, previous experimental studies have demonstrated that the biological activity of activating TSHR mutants may vary depending on the cellular context, indicating that constitutive cAMP activity alone does not fully determine clinical severity [19]. In the present study, functional analyses demonstrated that D633N increased basal cAMP activity compared with wild‐type TSHR, but its activity was lower than that of previously reported activating variants, such as V656F, D633E, and D633Y. These findings suggest that differences in basal receptor activation may partly contribute to variation in clinical presentation. The relationship between TSHR variants and their functional and clinical characteristics is summarized in Figure 7.

In addition to receptor‐intrinsic activity, environmental factors may also influence the clinical expression of activating TSHR variants. In particular, iodine intake has been suggested as a potential modifier of thyroid enlargement and hyperthyroidism. Because dietary iodine intake is relatively high in Japan, environmental iodine exposure may have contributed to the delayed clinical manifestation of hyperthyroidism in this patient [20].

Nonautoimmune hyperthyroidism is generally managed using treatment strategies similar to those used for Graves’ disease, including ATDs, RI therapy, or surgery [4]. In the present case, potassium iodide monotherapy was initially selected because of the patient’s strong concern regarding the potential adverse effects of ATDs. However, thyroid hormone levels did not sufficiently improve, possibly due to iodine‐induced thyrotoxicosis associated with the large goiter. RI therapy was subsequently performed, but the patient ultimately required a total thyroidectomy because of the large goiter, worsening destructive thyroiditis following RI therapy, advanced age, and the presence of heart failure. In retrospect, earlier treatment with ATDs followed by definitive therapy, such as surgery, might have been preferable. Recent studies have also suggested that potassium iodide therapy may exacerbate thyrotoxicosis in patients with large goiters or autonomous thyroid disease [21], and iodide monotherapy should therefore be used cautiously in such patients.

Earlier genetic testing might also have influenced clinical management by raising suspicion of nonautoimmune hyperthyroidism and favoring earlier consideration of definitive surgical treatment rather than RI therapy. Although RI therapy is recommended in the American Thyroid Association guidelines for patients with hyperthyroidism complicated by cardiovascular disease [22], its efficacy may be limited in patients with large goiters, in whom surgical treatment is often considered a more appropriate definitive therapy [23]. These findings, combined with the clinical course of our case, suggest that in elderly patients with large goiters and antibody‐negative hyperthyroidism, iodine‐based therapy and RI should be used with caution, and earlier consideration of definitive surgical management may be warranted.

Several limitations should be acknowledged. First, familial genetic testing was not performed, limiting the assessment of penetrance and intrafamilial phenotypic variability. Testing was not conducted because the patient’s parents had already died, and the patient declined genetic testing or clinical evaluation of her children. If asymptomatic relatives carry the same variant, they might still be at an increased risk of developing thyroid dysfunction or cardiovascular complications later in life. Second, TSH‐stimulated cAMP responses were not evaluated. Therefore, functional characterization of the D633N variant in this study was limited to basal receptor activity. In Graves’ disease, cAMP generation is driven by stimulation of the TSHR by TSH or TSHR–TSAbs [24]. In contrast, elevated basal cAMP activity in the absence of ligand suggests receptor‐intrinsic activation, as observed in activating TSHR variants. Previous studies have also reported that such variants exhibit increased basal cAMP activity with a blunted or plateaued response to TSH stimulation, and the present variant may share similar functional properties [19].

In conclusion, this case expands the spectrum of activating TSHR mutations associated with nonautoimmune hyperthyroidism. Nonautoimmune hyperthyroidism should be considered in patients with antibody‐negative hyperthyroidism, even in elderly individuals without a known family history.

## Funding

The authors received no specific funding for this work.

## Consent

The patient provided written informed consent, and this study was approved by the University of Tsukuba Hospital Ethics Committee (Approval Number R03‐130).

## Conflicts of Interest

The authors declare no conflicts of interest.

## Data Availability

The data that support the findings of this study are available from the corresponding author upon reasonable request.
